# Oligometastatic GIST: Impact of Treatment Modalities and Metastatic Distribution on Overall Survival †

**DOI:** 10.3390/cancers18162622

**Published:** 2026-08-14

**Authors:** Winston Hayes Pearce, Nikita Sharma, Leonardo Simonelli, Maiya-Mari Messina, Tanner Hill, Yu-Cherng Channing Chang, Mohammad Saleh, Emily Jonczak, Andrew E. Rosenberg, Nipun Merchant, Alan S. Livingstone, Dido Franceschi, Caitlin A. Hester, Julie Grossman, Francesco Alessandrino

**Affiliations:** 1 Miller School of Medicine, University of Miami, Miami, FL 33136, USA; 2 Department of Radiology, Miller School of Medicine, University of Miami, Miami, FL 33136, USA; 3 Department of Medicine, Miller School of Medicine, University of Miami, Miami, FL 33136, USA; 4 Sylvester Comprehensive Cancer Center, Miami, FL 33136, USA; 5 Department of Pathology, Miller School of Medicine, University of Miami, Miami, FL 33136, USA; 6 Department of Surgery, Miller School of Medicine, University of Miami, Miami, FL 33136, USA

**Keywords:** oligometastasis, gastrointestinal stromal tumor, metastasectomy, sarcoma, metastasis

## Abstract

**Background/Objectives**: Oligometastatic GIST, defined in this study as five or fewer metastatic lesions confined to a single organ, represents a distinct subset with a lower metastatic burden than widely metastatic disease and may have different prognostic and therapeutic considerations. **Methods**: We reviewed biopsy-proven oligometastatic GIST diagnosed between August 1998 and June 2025 from a prospectively maintained institutional database, collecting genomic, metastatic, treatment, ethnicity, and survival data. Overall survival (OS), calculated from diagnosis of oligometastatic disease, was estimated by Kaplan–Meier analysis and compared using the log-rank test. Hazard ratios (HRs) and 95% confidence intervals (CIs) were estimated using Cox proportional hazards regression. **Results**: Of 525 subjects with GIST in our database, 96 (18.3%) had oligometastatic GIST (median age: 54 years; median OS from diagnosis of oligometastatic disease: 9.77 years). Most tested tumors were KIT-positive (91.6%), predominantly harboring KIT exon 11 alterations (69.5%). The most common metastatic sites were the liver (*n* = 44) and peritoneum (*n* = 37). Overall, 58 patients underwent surgery, including 55 cytoreductive procedures and 3 emergent operations for bleeding or obstruction. Among the cytoreductive procedures, indications included multifocal disease after tyrosine kinase inhibitor (TKI) response (*n* = 21), unifocal disease after TKI response (*n* = 18), unifocal progression on TKI (*n* = 13), and equivocal or undocumented TKI response (*n* = 3). Overall survival did not differ by liver versus peritoneal metastases (HR: 1.27; 95% CI, 0.59–2.70; *p* = 0.540) or Hispanic versus non-Hispanic ethnicity (HR: 0.81; 95% CI, 0.34–1.90; *p* = 0.624). Cytoreductive surgery combined with systemic therapy was associated with longer overall survival than systemic therapy alone (10.8 vs. 7.4 years; HR: 0.44; 95% CI, 0.22–0.89; *p* = 0.019). **Conclusions**: In this retrospective cohort, cytoreductive surgery combined with systemic therapy was associated with longer overall survival than systemic therapy alone.

## Simple Summary

Oligometastatic gastrointestinal stromal tumor (GIST) represents a distinct clinical state in which the role of metastasis-directed surgery remains uncertain. In this study, oligometastatic GIST was defined as five or fewer metastatic lesions confined to a single organ. We retrospectively reviewed 96 patients with biopsy-proven oligometastatic GIST from a prospectively maintained institutional database spanning 1998 to 2025. Clinical, genomic, treatment, and survival data were analyzed to identify factors associated with outcomes. Patients treated with cytoreductive surgery in addition to tyrosine kinase inhibitor therapy had longer median overall survival than those receiving systemic therapy alone (10.8 vs. 7.4 years; HR: 0.44; 95% CI, 0.22–0.89; *p* = 0.019). These hypothesis-generating findings support prospective, multi-institutional studies to better define patient selection, treatment sequencing, and the role of metastasis-directed surgery in oligometastatic GIST.

## 1. Introduction

Gastrointestinal stromal tumor (GIST) is the most common mesenchymal malignancy of the gastrointestinal tract and demonstrates a broad spectrum of clinical behavior, ranging from indolent localized disease to aggressive metastatic progression. The five-year relative survival rate ranges from 96% for localized disease to 87% and 57% for regional and distant metastatic disease, respectively [1].

Treatment of oligometastatic GIST, defined in this study as the presence of five or fewer metastatic lesions confined to a single organ, remains challenging because of substantial heterogeneity in tumor biology, patterns of metastatic spread, and responsiveness to systemic therapy [2,3]. This more restrictive definition was selected to reduce biologic heterogeneity within the study cohort, although it may limit generalizability compared with broader consensus definitions. Emerging evidence across multiple metastatic solid tumors supports oligometastatic disease as a clinically distinct state associated with more favorable outcomes, rather than merely an early phase of disseminated disease [2]. This evolving paradigm has prompted increasing interest in evaluating survival outcomes and the potential role of locally directed therapies, including surgical cytoreduction, in patients with oligometastatic GIST [4,5]. Advances in minimally invasive surgical techniques have expanded treatment options for selected patients with localized GIST; however, the role of these approaches in oligometastatic disease remains incompletely defined [6,7]. Prior institutional experiences and retrospective cohort studies have suggested that patients with oligometastatic GIST may experience more favorable survival outcomes than those with more extensive metastatic disease [3].

Current National Comprehensive Cancer Network guidelines emphasize tyrosine kinase inhibitor (TKI) therapy as the cornerstone of management for metastatic GIST; however, optimal treatment strategies for oligometastatic disease are not well established [8]. In other metastatic malignancies, such as breast cancer and non-small cell lung cancer, multiple studies have demonstrated a survival benefit for select patients with a low metastatic burden treated with surgical resection or other local therapies in combination with systemic treatment [9,10]. Similar survival associations have been described in oligometastatic soft tissue sarcomas (STS) [11,12]. Several retrospective studies and reviews have examined the potential impact of surgery in addition to systemic therapy in oligometastatic GIST [3,4,5]. A 2021 retrospective cohort study by Walls et al. demonstrated that a significant proportion of patients with oligometastatic STS experienced durable disease control with a multimodal approach, with metastasectomy being the most common locally directed intervention [11]. Despite these developments, patient selection criteria and the role of metastasis-directed interventions in oligometastatic GIST remain unclear, and there is no standardized consensus regarding the optimal timing, sequencing, or integration of local therapies with systemic treatment. As a result, management decisions are often individualized and influenced by clinician judgment and institutional practice patterns [2,3,4,5].

This article is a revised and expanded version of an abstract entitled Oligometastatic gastrointestinal stromal tumor: Association between treatment type, metastatic pattern, and survival, which was published online at the American Society of Clinical Oncology 2025 Annual Meeting [13]. Substantially expanding upon our preliminary data, in this single-center retrospective study, we sought to further characterize outcomes in patients with oligometastatic GIST by evaluating whether overall survival (OS) differs according to metastatic site, ethnicity, and treatment modality, with the goal of identifying factors that may inform risk stratification and guide multidisciplinary management in this patient population [13].

## 2. Materials and Methods

### 2.1. Patient Population

This retrospective study was conducted at a single tertiary referral center, using Health Insurance Portability and Accountability Act-compliant data under Institutional Review Board approval. We queried a prospectively maintained pan-sarcoma database to identify patients with biopsy-proven GIST diagnosed between August 1998 and June 2025. Eligible subjects met the following criteria: (1) age ≥18 years at the time of diagnosis, (2) histopathologic confirmation of GIST by a sarcoma pathologist, and (3) oligometastatic disease defined as the presence of five or fewer metastatic lesions confined to a single organ, based on radiologic review. This more restrictive definition was selected to assess a biologically homogeneous cohort with a limited metastatic burden, recognizing that broader consensus definitions may permit limited metastatic involvement of multiple organs. Application of these criteria yielded the final study cohort.

### 2.2. Clinical and Histopathologic Data

Electronic medical records were reviewed to extract demographic, clinical, and pathologic variables, including age at diagnosis, sex, self-reported ethnicity, date of diagnosis, primary tumor site, mutational status via next-generation sequencing, number and sites of metastatic disease, and treatment history. Treatment modalities were categorized as systemic therapy alone or systemic therapy combined with surgical intervention. OS was defined as the interval from the date of oligometastatic diagnosis to death from any cause. Patients alive at last follow-up were censored.

### 2.3. Assessment of Oligometastatic Disease

Oligometastatic disease was defined as the presence of five or fewer metastatic lesions confined to a single organ at the time of metastatic diagnosis. To identify eligible patients, contrast-enhanced computed tomography (CT) of the chest, abdomen, and pelvis, fluorodeoxyglucose F-18 (FDG) positron emission tomography (PET)/CT, and magnetic resonance imaging (MRI) studies obtained from the time of diagnosis through the last available follow-up were independently reviewed by two radiologists-in-training (Y.C.C., M.S.) and subsequently confirmed by a fellowship-trained diagnostic radiologist with more than 5 years of experience in sarcoma imaging (F.A.). Eligibility for the study was determined based on imaging at the time of initial metastatic diagnosis; subsequent imaging was reviewed to confirm lesion characterization and disease progression but did not alter baseline oligometastatic classification.

The date of the first imaging examination demonstrating metastatic disease or recurrence was recorded, together with the involved metastatic sites, as previously described [14,15]. Lymph node metastases were defined by a short-axis diameter of at least 1.5 cm, FDG avidity, histopathologic confirmation, or greater than 20% interval increase in short-axis diameter on follow-up imaging. Following curative treatment, newly identified lesions were classified as recurrent disease if confirmed histopathologically or if they demonstrated unequivocal progression according to RECIST 1.1 criteria [16]. Lesions identified before curative treatment were classified as metastatic when supported by histopathology or unequivocal interval growth on serial imaging, consistent with RECIST 1.1 guidelines [16].

For confluent metastases, including peritoneal metastatic deposits, imaging findings were reviewed jointly and resolved by consensus among the three readers. Confluent metastatic deposits were counted as a single lesion when they represented a contiguous focus of disease without intervening normal tissue, whereas spatially distinct foci separated by uninvolved tissue were counted as separate metastatic lesions, as detailed in RECIST 1.1 guidelines, even if located in the same peritoneal region [16].

### 2.4. Treatment

The timing and sequencing of systemic therapy (TKI) and surgical intervention were recorded when applicable. Surgical history was stratified into predefined categories reflecting surgical intervention for oligometastatic disease, including surgery for multifocal or unifocal disease following TKI response, surgery for unifocal progression with poor TKI response, and surgery for bleeding or obstruction. The timing and sequencing of non-surgical metastasis-directed local therapies such as radioembolization were documented as well.

### 2.5. Statistical Analysis

Associations between metastatic sites (liver, peritoneum, lymph nodes, stomach, colon, lung, retroperitoneum, ovary, spleen, pelvis, and abdominal wall) and surgical history were assessed using chi-square tests. Overall survival (OS) was calculated from the date of oligometastatic disease diagnosis to death or last follow-up, including patients who subsequently progressed to widespread metastatic disease. Median follow-up was estimated using the reverse Kaplan–Meier method. OS was estimated using the Kaplan–Meier method and compared according to metastatic site, treatment strategy, and Hispanic versus non-Hispanic ethnicity using the log-rank test. Hazard ratios (HRs) and 95% confidence intervals (CIs) were estimated using Cox proportional hazards regression. Statistical significance was defined as a two-sided *p* < 0.05.

Multivariable Cox proportional hazards regression was performed to evaluate the association between cytoreductive surgery and overall survival while adjusting for potential confounders. The model was intentionally restricted to a limited number of clinically relevant covariates to minimize overfitting and preserve model stability. Age at diagnosis and metastatic site (liver versus peritoneum) were selected because of their established clinical relevance and potential to confound the association between cytoreductive surgery and overall survival. Sensitivity analyses were performed post hoc by repeating the survival analyses after exclusion of patients with SDH-deficient GIST and, separately, after exclusion of patients who underwent radioembolization. The SDH-deficient sensitivity analysis was performed because of the distinct biological behavior of this molecular subtype. The radioembolization sensitivity analysis was performed because radioembolization was not mutually exclusive with either the cytoreductive surgery plus systemic therapy group or the systemic therapy-alone group, and the limited number of treated patients precluded separate survival analysis. A time-dependent Cox proportional hazards model incorporating surgery as a time-varying covariate was performed to evaluate the association between surgical treatment and overall survival while accounting for the timing of surgery. All statistical analyses were performed using R software (version 4.4.3).

## 3. Results

### 3.1. Patient Characteristics

Of 525 subjects with GIST identified in the pan-sarcoma database, 96 patients (18.3%) met criteria for oligometastatic GIST. The median age at diagnosis was 54 years (range, 26–79 years), with a male-to-female ratio of 1.04:1. The median overall survival (OS) from oligometastatic diagnosis was 9.77 years. During follow-up, 33 patients (34.4%) died. Twenty patients (20.8%) self-identified as Hispanic. No statistically significant difference in OS was observed between Hispanic and non-Hispanic patients (HR: 0.807; 95% CI, 0.343–1.900; *p* = 0.624). The baseline characteristics of the retrospective cohort are presented in Table 1.

### 3.2. Oligometastatic Sites

**Figure 1:**
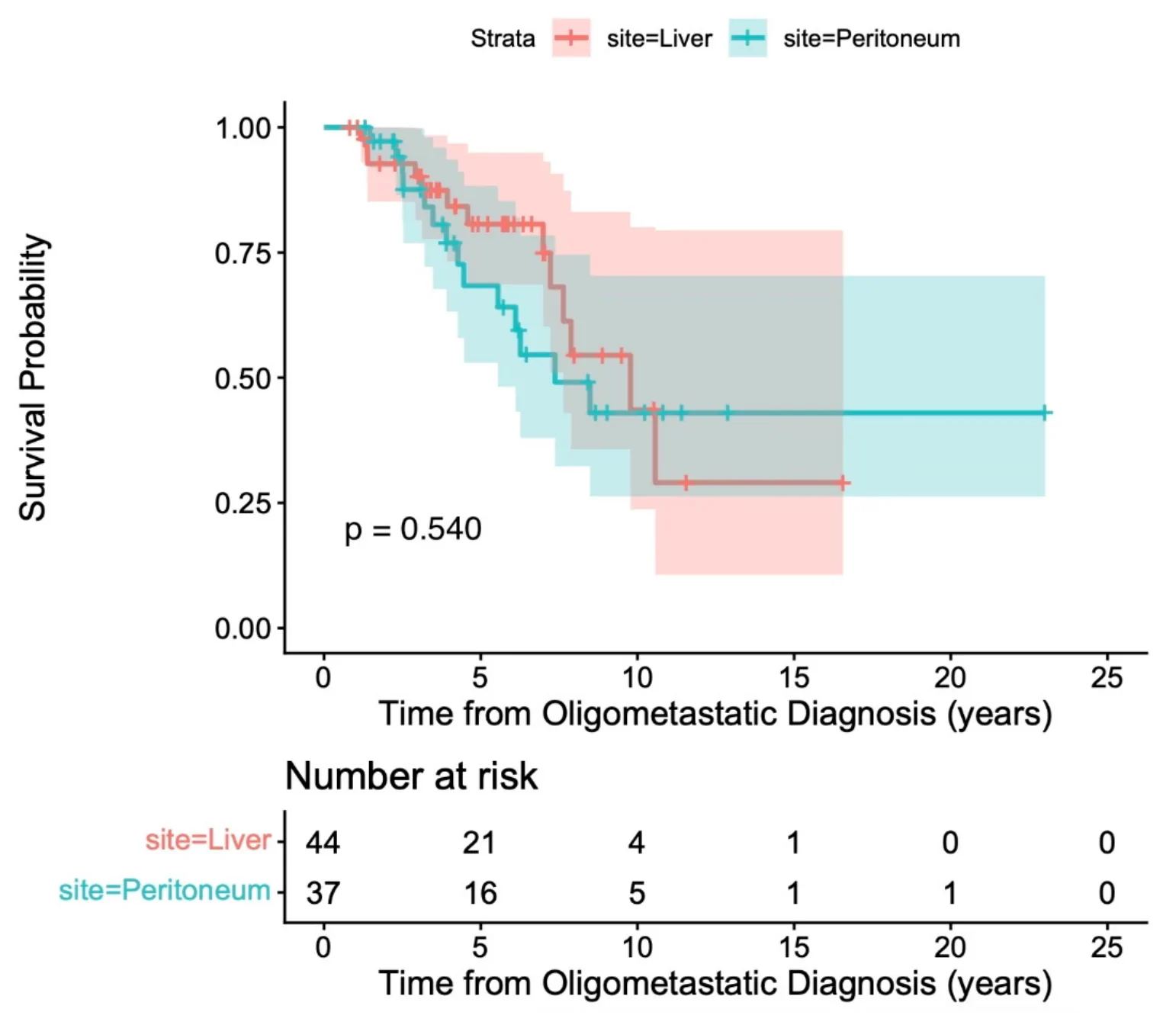
Kaplan–Meier overall survival curves comparing patients with liver versus peritoneal oligometastatic GIST. Patients with liver metastases are shown in red and patients with peritoneal metastases in blue. Shaded regions represent 95% confidence intervals, and tick marks indicate censored observations. Median overall survival was 9.78 years for patients with liver metastases and 7.37 years for patients with peritoneal metastases. No statistically significant difference in overall survival was observed between metastatic sites (HR: 1.27; 95% CI, 0.59–2.70; *p* = 0.540). Numbers at risk over time are displayed below the plot.

The liver was the most common site of oligometastatic disease (*n* = 44), followed by the peritoneum (*n* = 37) and lymph nodes (*n* = 5). Median overall survival was 9.78 years among patients with liver metastases and 7.37 years among those with peritoneal metastases. Kaplan–Meier analysis demonstrated no significant difference in overall survival between patients with liver and peritoneal oligometastatic disease (Figure 1). This finding was consistent with Cox proportional hazards regression, which likewise showed no significant association between metastatic site and overall survival (HR: 1.27; 95% CI, 0.59–2.70; *p* = 0.540).

### 3.3. Mutational Status

Among the 96 patients in the study cohort, 95 (99.0%) underwent KIT testing, of whom 87 (91.6%) had KIT-positive tumors. KIT exon 11 alterations were the most common (66/95, 69.5%), followed by exon 9 (15/95, 15.8%) and exon 17 (7/95, 7.4%). Individual KIT exon categories were not mutually exclusive. PDGFRA alterations were identified in 6 of 71 tested patients (8.5%), whereas SDH deficiency was identified in 5 of 70 tested patients (7.1%) (Table 1). Because molecular testing was not uniformly available and mutation-specific subgroups were small, the study was not powered to evaluate associations between individual molecular subtypes and overall survival.

### 3.4. Surgical History and Overall Survival

Of the 96 patients, 55 (57.3%) underwent cytoreductive surgery in addition to systemic therapy, whereas 38 (39.6%) received systemic therapy alone. Among patients undergoing cytoreductive surgery, indications included multifocal disease following response to TKI therapy in 21 patients (21.9% of the overall cohort), unifocal disease following TKI response in 18 (18.8%), unifocal progression with poor TKI response in 13 (13.5%), and equivocal or undocumented TKI response in 3 (3.1%). Three additional patients (3.1%) underwent emergent surgery for bleeding or obstruction and were excluded from the comparative survival analysis because these procedures were performed for symptom control rather than with cytoreductive intent. The proportion of patients undergoing cytoreductive surgery did not differ between patients with liver and peritoneal oligometastatic disease (24/44 [54.5%] vs. 21/37 [56.8%]; χ^2^ *p* = 0.842).

**Figure 2:**
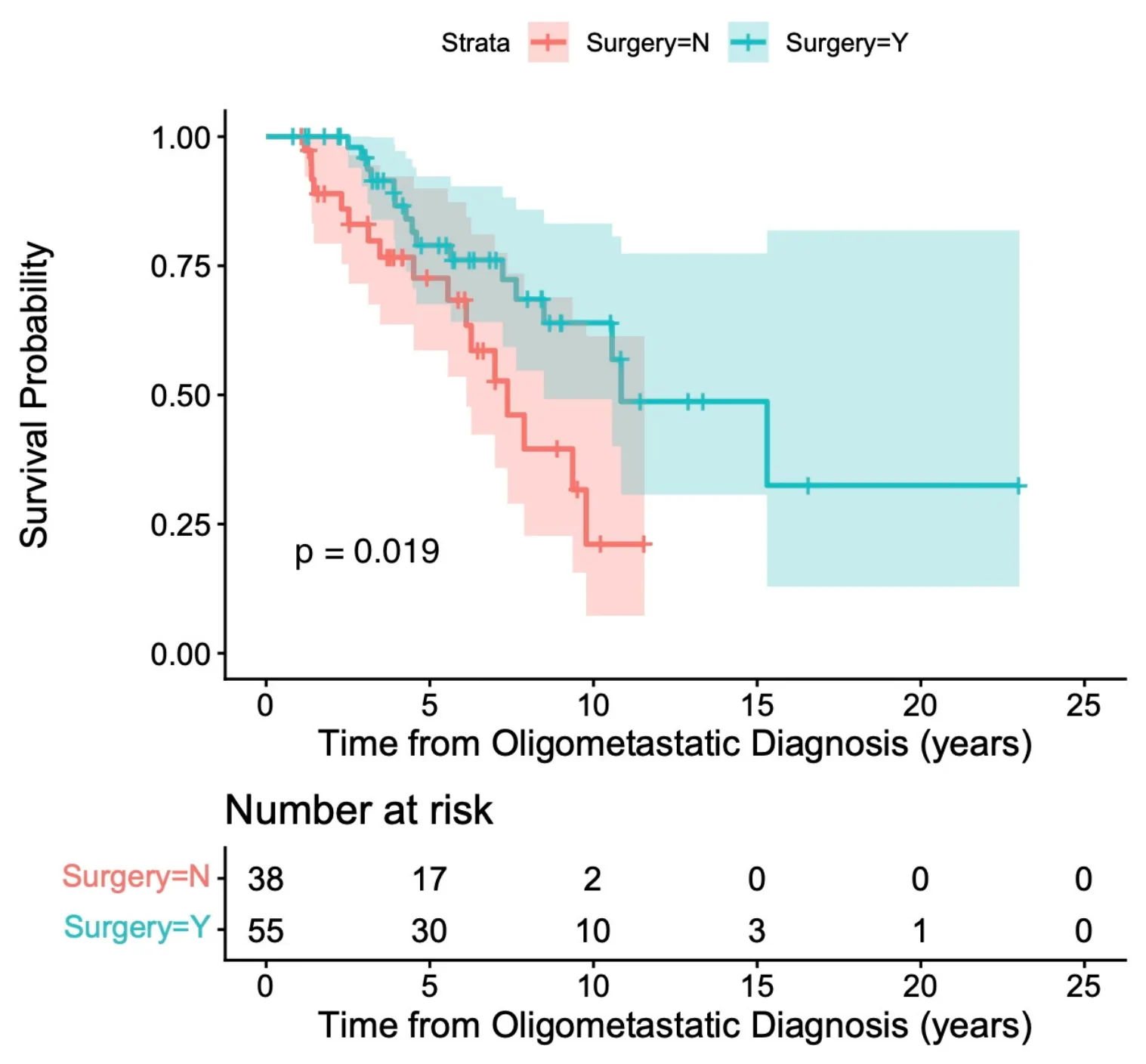
Kaplan–Meier overall survival curves comparing patients treated with cytoreductive surgery plus systemic therapy versus systemic therapy alone for oligometastatic gastrointestinal stromal tumor (GIST). Patients treated with systemic therapy alone are shown in red, while those treated with cytoreductive surgery plus systemic therapy are shown in blue. Shaded regions represent 95% confidence intervals, and tick marks indicate censored observations. Cytoreductive surgery combined with systemic therapy was associated with longer overall survival than systemic therapy alone (HR: 0.44; 95% CI, 0.22–0.89; *p* = 0.019). Numbers at risk over time are displayed below the plot.

Patients who underwent cytoreductive surgery in combination with systemic therapy demonstrated longer overall survival than those treated with systemic therapy alone. Median overall survival from the time of oligometastatic diagnosis was 10.84 years in the cytoreductive surgery group and 7.37 years in the systemic therapy-alone group (HR: 0.44; 95% CI, 0.22–0.89; *p* = 0.019) (Figure 2).

### 3.5. Multivariable Analysis of Cytoreductive Surgical History, Age, and Metastatic Site

Because of the limited number of observed deaths, a multivariable Cox proportional hazards model was intentionally restricted to a small number of clinically relevant covariates to minimize overfitting. Age at diagnosis and metastatic site (liver versus peritoneum), the two most common metastatic sites in the cohort, were selected a priori because of their established clinical relevance and potential to confound the association between cytoreductive surgery and overall survival. In this model, cytoreductive surgery remained associated with overall survival, although the association did not reach statistical significance (adjusted HR: 0.49; 95% CI, 0.22–1.07; *p* = 0.072). Neither age at diagnosis (adjusted HR: 1.01; 95% CI, 0.98–1.04; *p* = 0.422) nor metastatic site (peritoneum versus liver; adjusted HR: 1.45; 95% CI, 0.67–3.12; *p* = 0.348) was independently associated with overall survival. Assessment of Schoenfeld residuals demonstrated no evidence of violation of the proportional hazards assumption for any covariate or for the model overall (global *p* = 0.81), supporting the validity of the Cox proportional hazards model.

### 3.6. Sensitivity Analysis Excluding Patients Who Underwent Radioembolization

Given that radioembolization was not mutually exclusive with either the cytoreductive surgery plus systemic therapy group or the systemic therapy-alone group, and because the number of patients who underwent radioembolization was too small to permit a meaningful independent Kaplan–Meier analysis, a sensitivity analysis excluding these patients was performed. Following exclusion, cytoreductive surgery remained significantly associated with longer overall survival than systemic therapy alone. Median overall survival was 10.84 years in the cytoreductive surgery group versus 6.99 years in the systemic therapy-alone group (HR: 0.33; 95% CI, 0.15–0.73; *p* = 0.006). The corresponding Kaplan–Meier analysis was also significant by log-rank testing (*p* = 0.004), indicating that a similar survival association persisted after patients treated with radioembolization were excluded.

### 3.7. Sensitivity Analysis for Influence of SDH Deficiency

Because SDH-deficient GIST represents a biologically distinct molecular subtype with a different clinical course, a sensitivity analysis excluding these patients was performed. After exclusion of SDH-deficient tumors, cytoreductive surgery remained associated with a similar magnitude of longer overall survival compared with systemic therapy alone, although the association was attenuated and no longer reached statistical significance in the Cox proportional hazards model (median overall survival, 10.84 vs. 7.37 years; HR: 0.50; 95% CI, 0.25–1.01; *p* = 0.053). The corresponding Kaplan–Meier analysis remained significant by log-rank testing (*p* = 0.049), suggesting that the observed survival association was not solely attributable to the inclusion of patients with SDH-deficient GIST.

### 3.8. Time-Dependent Cox Proportional Hazards Model for Cytoreductive Surgery Status

Surgical intervention was modeled as a time-dependent exposure in a Cox proportional hazards model to account for the timing of surgery relative to the diagnosis of oligometastatic disease. The direction of effect remained consistent with the primary analysis, with surgery remaining associated with a lower hazard of death, although the association was not statistically significant (HR: 0.70; 95% CI, 0.34–1.42; *p* = 0.32).

### 3.9. Median Follow-Up Time via Reverse Kaplan–Meier Method

Median follow-up, estimated using the reverse Kaplan–Meier method, was 6.64 years (95% CI, 5.78–8.90) for the overall cohort. Median follow-up was 7.04 years (95% CI, 5.72–9.02) among patients undergoing cytoreductive surgery and 6.48 years (95% CI, 4.91–not reached) among patients receiving systemic therapy alone. There was no significant difference in follow-up duration between treatment groups (log-rank *p* = 0.50).

## 4. Discussion

In this single-institution retrospective analysis, patients with oligometastatic GIST represented a distinct clinical subset and demonstrated prolonged median overall survival compared with historical expectations for advanced disease. Within this cohort, metastasis-directed surgery in combination with systemic therapy was associated with longer survival compared with systemic therapy alone. Specifically, median overall survival from the time of oligometastatic diagnosis was 10.8 years in patients who underwent surgery, compared with 7.4 years in those managed without surgery.

It has been proposed that cytoreductive approaches may influence outcomes by reducing overall tumor burden, limiting clonal heterogeneity, and delaying the emergence of resistant tumor clones. In 2018, Fairweather et al. reported an association between cytoreductive surgery and longer survival among patients with metastatic GIST who demonstrated stable disease on TKI, while observing no significant association among patients with multifocal progressive disease [5]. Similarly, Chang et al. reported that cytoreductive surgery combined with imatinib was associated with higher treatment response rates and prolonged progression-free survival compared with systemic therapy alone, although no statistically significant difference in overall survival was observed [17]. Collectively, these and other retrospective studies have contributed to the evolving hypothesis that surgical intervention may have a role in carefully selected patients with limited metastatic burden and controlled systemic disease [5,17,18,19,20,21]. Consistent with these observations, Brink et al. also reported favorable outcomes among selected patients undergoing local treatment for metastatic GIST, while emphasizing that the retrospective design of available studies makes it difficult to distinguish the effects of local therapy from the more favorable disease characteristics of patients selected for intervention [22].

The potential benefit of metastasis-directed therapy has also been explored across several other solid malignancies, with increasing evidence supporting multidisciplinary local consolidative approaches for carefully selected patients with oligometastatic disease [21]. In oligometastatic non-small cell lung cancer, randomized studies evaluating local consolidative therapy have reported improvements in progression-free survival and, in selected patients, OS, supporting the broader role of metastasis-directed treatment in appropriately selected patients [23,24,25]. Similarly, emerging evidence in oligometastatic breast cancer suggests that carefully selected patients may experience prolonged disease control following metastasis-directed local therapy, although prospective evidence remains limited and patient selection remains critical [26,27,28]. While these findings cannot be directly extrapolated to GIST because of distinct tumor biology and patterns of systemic therapy response, they provide a conceptual framework supporting further investigation of local treatment strategies in carefully selected patients with oligometastatic disease.

Appropriate patient selection remains central to the consideration of metastasis-directed surgery in oligometastatic GIST. Factors that have been proposed to influence treatment decisions include response to TKI, overall tumor biology, technical resectability, patient performance status, and multidisciplinary evaluation within experienced sarcoma centers [22,29]. These considerations likely contributed to surgical selection in the present cohort and represent potential sources of residual confounding that cannot be fully accounted for in a retrospective analysis. In addition, socioeconomic and healthcare access factors have also been associated with survival outcomes among patients with metastatic GIST, further emphasizing that observed differences in outcomes may reflect both measured and unmeasured patient characteristics rather than treatment alone [30]. Consequently, the association between metastasis-directed surgery and overall survival observed in the present study should be interpreted cautiously and considered hypothesis-generating rather than evidence of a causal treatment effect.

Metastatic patterns in this cohort were consistent with prior studies, with the liver and peritoneum representing the most common sites of disease spread [2,13]. However, metastatic site was not independently associated with overall survival, suggesting that disease biology and treatment response may be more important determinants of prognosis than anatomic location alone within the oligometastatic setting. Similarly, no significant survival differences were observed according to ethnicity, although interpretation is limited by the single-institution design.

Additional sensitivity analyses yielded effect estimates similar to the primary findings. Exclusion of patients with SDH-deficient GIST yielded a similar effect estimate despite attenuation of statistical significance, indicating that the observed survival association was not solely attributable to this biologically distinct subtype. SDH-deficient GIST represents a rare molecular subtype characterized by unique tumor biology, relative resistance to conventional TKI, and often a more indolent clinical course [31,32]. Data specifically evaluating oligometastatic SDH-deficient GIST remain scarce, and future multicenter collaborative studies will be necessary to determine whether optimal patient selection and the role of metastasis-directed therapy differ within this uncommon subgroup. Similarly, excluding patients who underwent radioembolization did not materially alter the observed association between surgery and overall survival, although radioembolization has emerged as a potential treatment option for selected patients with liver-dominant metastatic GIST [33,34].

Several important limitations should be considered when interpreting these findings. First, the retrospective, single-institution design introduces the potential for selection bias, as patients selected for surgery may have had more favorable disease biology, superior performance status, greater response to TKI, and lower disease burden. Although multivariable adjustment and multiple sensitivity analyses were performed, residual confounding inherent to retrospective observational studies cannot be excluded.

To further address immortal-time bias, surgery was modeled as a time-dependent exposure. Although the magnitude of the association was attenuated and no longer reached statistical significance, the direction of effect remained consistent with the primary analysis. Notably, nearly half of surgical patients (27/55, 49.1%) underwent resection within the first year following oligometastatic diagnosis, underscoring the importance of accounting for treatment timing in survival analyses. Median follow-up, estimated using the reverse Kaplan–Meier method, did not differ significantly between treatment groups, suggesting that differential follow-up is unlikely to account for the observed survival association.

Detailed operative variables, including surgical approach, extent of resection, margin status, and perioperative morbidity, were not uniformly available because many patients were referred to our tertiary sarcoma center after undergoing surgery at outside institutions, where operative reports and perioperative outcomes were frequently unavailable or incomplete. Consequently, these factors could not be evaluated.

Furthermore, molecular data were not uniformly available for all patients, limiting detailed genotype–phenotype correlations and survival analyses of individual KIT exon mutations. Additionally, the relatively small size of the oligometastatic cohort restricted statistical power for subgroup and interaction analyses and precluded more comprehensive multivariable adjustment incorporating molecular characteristics.

Finally, some patients with limited disease may have been excluded based on our definition of oligometastatic disease, limiting our results to a population of patients with single-organ oligometastatic disease.

## 5. Conclusions

In this single-institution retrospective study, cytoreductive surgery in combination with systemic therapy was associated with longer overall survival than systemic therapy alone among patients with oligometastatic GIST. These findings should be interpreted cautiously given the potential for selection bias, immortal-time bias, and residual confounding inherent to retrospective observational studies. Accordingly, the observed association should be considered hypothesis-generating rather than evidence of a causal treatment effect. Prospective, multicenter studies are warranted to validate these findings and to better define the role, timing, and patient selection for metastasis-directed surgery in oligometastatic GIST.

## Figures and Tables

**Table 1:** Baseline Characteristics of Patients With Oligometastatic GIST (N = 96).

Characteristic	Oligometastatic GIST (N = 96)
**Age at diagnosis**	
Median age, years	54 [range: 26–79 years]
**Sex**	
Male	49 (51.0%)
Female	47 (49.0%)
**Ethnicity**	
Hispanic	20 (20.8%)
Non-Hispanic	76 (79.2%)
**Surgical history**	
No surgery	38 (39.6%)
Surgery for multifocal disease after TKI response	21 (21.9%)
Surgery for unifocal disease after TKI response	18 (18.8%)
Surgery for unifocal progression with poor TKI response	13 (13.5%)
Equivocal or undocumented TKI response	3 (3.1%)
Surgery for bleeding or obstruction	3 (3.1%)
Radioembolization	7 (7.3%)
**KIT testing** ^a,^*	
Exon 11	66 (69.5%)
Exon 9	15 (15.8%)
Exon 17	7 (7.4%)
KIT-negative	8 (8.4%)
PDGFRA-positive ^b^	6 (8.5%)
SDH-deficient ^c^	5 (7.1%)
**Oligometastatic sites**	
Liver	44 (45.8%)
Peritoneum	37 (38.5%)
Lymph nodes	5 (5.2%)
Stomach	2 (2.1%)
Colon	2 (2.1%)
Lung	1 (1.0%)
Retroperitoneum	1 (1.0%)
Ovary	1 (1.0%)
Spleen	1 (1.0%)
Pelvis	1 (1.0%)
Abdominal wall	1 (1.0%)

Abbreviations: GIST—gastrointestinal stromal tumor, TKI—tyrosine kinase inhibitor, PDGFRA—platelet-derived growth factor receptor alpha, SDH—succinate dehydrogenase; ^a^ among 95 patients with documented KIT testing; ^b^ among 71 patients with documented PDGFRA testing; ^c^ among 70 patients with documented SDH testing; * KIT exon categories were not mutually exclusive.

## Data Availability

The data presented in this study are available on request from the corresponding author.

## Abbreviations

The following abbreviations are used in this manuscript:

GIST	Gastrointestinal stromal tumor
TKI	Tyrosine kinase inhibitor
STS	Soft tissue sarcoma
OS	Overall survival
CT	Computed tomography
FDG	Fluorodeoxyglucose F-18
PET	Positron emission tomography
MRI	Magnetic resonance imaging
CI	Confidence interval

## Appendix A

Additional members of the Bone and Soft Tissue Sarcomas Team of the Sylvester Comprehensive Cancer Center, University of Miami, Miami, FL.

Jonathan C. Trent ^1^, Gina Z. D’Amato ^1^, Steven Bialick ^1^, Emanuela Palmerini ^1^, Morgan Smith Mount ^1^, Solange Sierra ^1^, Alissette Naveda ^1^, Zulay Danli Chang ^1^, Aditi Dhir ^2^, Francis John Hornicek ^3^, H. Thomas Temple ^3^, Brooke M. Crawford ^3^, Erik John Geiger ^3^, Raphael Yechieli ^4^, Aaron H. Wolfson ^4^, Laura Michelle Freedman ^4^, Felipe Ferreira de Souza ^5^, Elizabeth Montgomery ^6^, Ty K. Subhawong ^7^, Patricia Castillo ^7^

^1^ Department of Medicine, University of Miami Miller School of Medicine, Miami, FL
^2^ Department of Pediatrics, University of Miami Miller School of Medicine, Miami, FL
^3^ Department of Orthopedics, University of Miami Miller School of Medicine, Miami, FL
^4^ Department of Radiation Oncology, University of Miami Miller School of Medicine, Miami, FL
^5^ Department of Interventional Radiology, University of Miami Miller School of Medicine, Miami, FL
^6^ Department of Pathology, University of Miami Miller School of Medicine, Miami, FL
^7^ Department of Radiology, University of Miami Miller School of Medicine, Miami, FL
